# Ice-man Down: Using Simulation to Practice the Safe Extrication of Collapsed Hockey Players in a Confined Space

**DOI:** 10.7759/cureus.2622

**Published:** 2018-05-14

**Authors:** Meryl Abrams, Dimitrios Papanagnou, Carlos Rodriguez, Joshua Rudner, Hyunjoo Lee, Simran Buttar, Ronald V Hall, Xiao Chi Zhang

**Affiliations:** 1 Department of Emergency Medicine, Thomas Jefferson University, Philadelphia, USA; 2 Department of Emergency Medicine, Thomas Jefferson University , Philadelphia, USA; 3 Emergency Medicine, Maimonides Medical Center

**Keywords:** emergency medicine, sport medicine, ice hockey, syncope, cervical spine injury, immobilization, team building

## Abstract

Sporting event emergencies are common among both spectators and players, with unique sets of challenges associated with patient extrication in unfamiliar and chaotic environments. It is critical for sports physicians and trainers to deliberately train and prepare for emergent situations with limited resources during athletic events. One of the most difficult, yet commonly encountered challenges is determining when and how to safely remove an injured player’s helmet and sporting equipment, particularly if a spinal injury is highly suspected.

We created a high-fidelity simulation case to practice the safe extrication of a hockey player who collapses on the bench in the player’s box, a space-restricted environment. The patient is a 25-year-old male hockey player who becomes unresponsive after a syncopal episode in the player’s box, and subsequently transferred to a medical center for further evaluation. Critical actions include extrication of the player at the scene, diagnosis of syncope, placement of the unconscious player on a backboard with cervical-spine precautions, removal of the player’s faceguard, removing the player off the ice, checking the electrocardiogram and glucose level, and transferring the player to a controlled environment. The learning objectives were to identify, evaluate, and manage the reversible causes of syncope, and demonstrate appropriate techniques for the optimal removal of sports equipment. Learner assessment was based on participation in the scenario and debriefing learners after the simulation.

Post-simulation debriefing revealed that participants highly appreciated practicing not-so-commonly encountered hockey-related emergencies. Athletic trainers and emergency providers were able to effectively practice their management of the unresponsive hockey player. The participants were also able to deliberately practice their teamwork and communications skills with their peers. Learning points include proper c-spine immobilization techniques in a tight space and indication for gear-removal in an unconscious patient.

As hockey continues to gain popularity, this simulation case will prepare athletic trainers and emergency providers to better address the reversible causes for syncope in hockey players, as well as safely and effectively extricate injured players from space-limiting sporting environments.

## Introduction

Sporting event emergencies are common among both spectators and players, with unique sets of challenges associated with patient extrication in unfamiliar and chaotic environments. Preparing and planning for medical emergencies at sporting events has been shown to improve the efficiency of providing appropriate care [1], and as a result, many sporting associations have developed disaster plans and medical guides for their personnel. It is, therefore, critical for sports physicians and trainers to deliberately train and prepare for emergent situations with limited resources during athletic events [2].

One of the most difficult, yet commonly encountered challenges is determining when and how to safely remove an injured player’s helmet and sporting equipment, particularly if a spinal injury is highly suspected. Spinal injuries are relatively common in athletes when compared to the general population, and may be the primary injury during a high-force mechanism. Ice hockey is a classic example of a high-velocity sport, in which a spinal injury may occur [1].

Following a traumatic event in fully-geared athletic players (i.e., hockey, football), all equipment should initially be kept in place, except for urgent access to the chest (for chest compressions and defibrillation) and the airway (for possible obstruction and/or ventilation). Pre-hospital helmet removal has the potential to hyper-extend the cervical spine. According to pre-hospital literature, in acute head-trauma events, a player’s helmet or shoulder pads should not be removed until the player has arrived at the hospital, except for the following emergent situations: unresponsiveness (or syncope), suspicion for intracranial injury, inability to access the airway after faceguard removal, or a loose/ill-fitting helmet that interferes with adequate spinal immobilization [3]. When evaluating a patient with syncope (defined as a transient loss of consciousness due to inadequate cerebral blood flow leading to decreased postural tone and collapse), medical personnel must be able to rapidly identify a syncopal event; assess the airway, breathing and circulation; and assess for reversible causes of the syncopal episode (i.e., hypoglycemia) before progressing to removing any gear and equipment [4].

**Figure 1:**
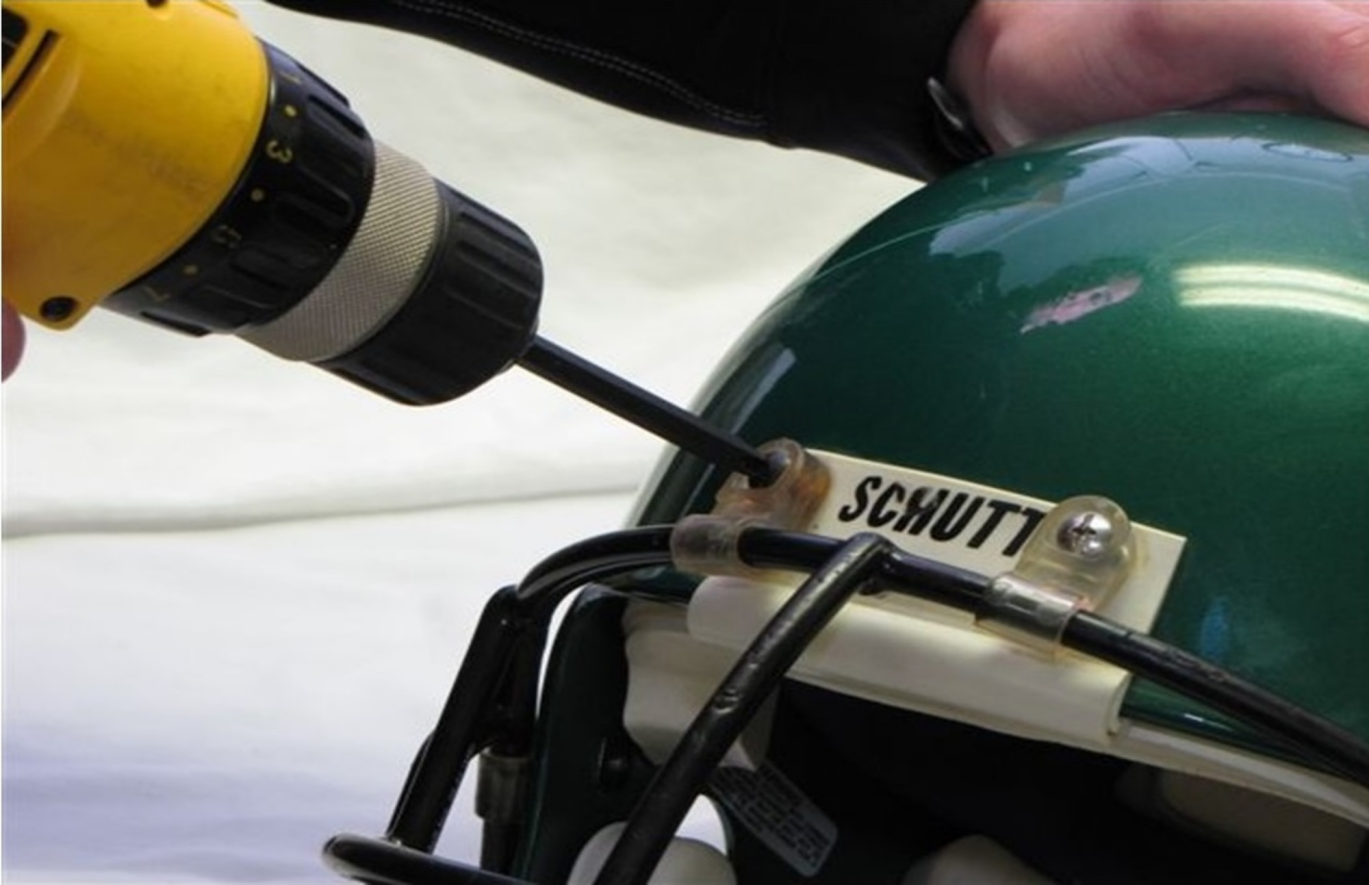
Faceguard removal Removal of the faceguard using an electric screwdriver.

Removal of the helmet and pads separately have been shown to result in increased sub-axial cervical spine lordosis, spinal cord impingement, and (potentially) quadriplegia [5-7]. If equipment must be moved, it is critical to remove them simultaneously through a regimented, practiced method using at least three to four trained professionals to limit cervical motion [2,5-6]. While many helmets have faceguards that can be easily retracted, the safest and most effective faceguard removal technique is following the steps below:

Step 1: Use an electric screwdriver to remove the screws connecting the faceguard to the helmet (Figure 1) [8-9].

Step 2: If the screwdriver is inadequate, consider using a cutting device for backup.

Step 3: Once the faceguard is removed, adequate airway exposure can be achieved without removing the helmet.

Blind invasive airway interventions such as the laryngeal mask airway (LMA) or esophageal tracheal double-lumen airway (i.e., Combitube airway) can be utilized without removing the faceguard during airway emergencies. However, if an endotracheal tube is indicated, the faceguard must be removed [10].

**Figure 2:**
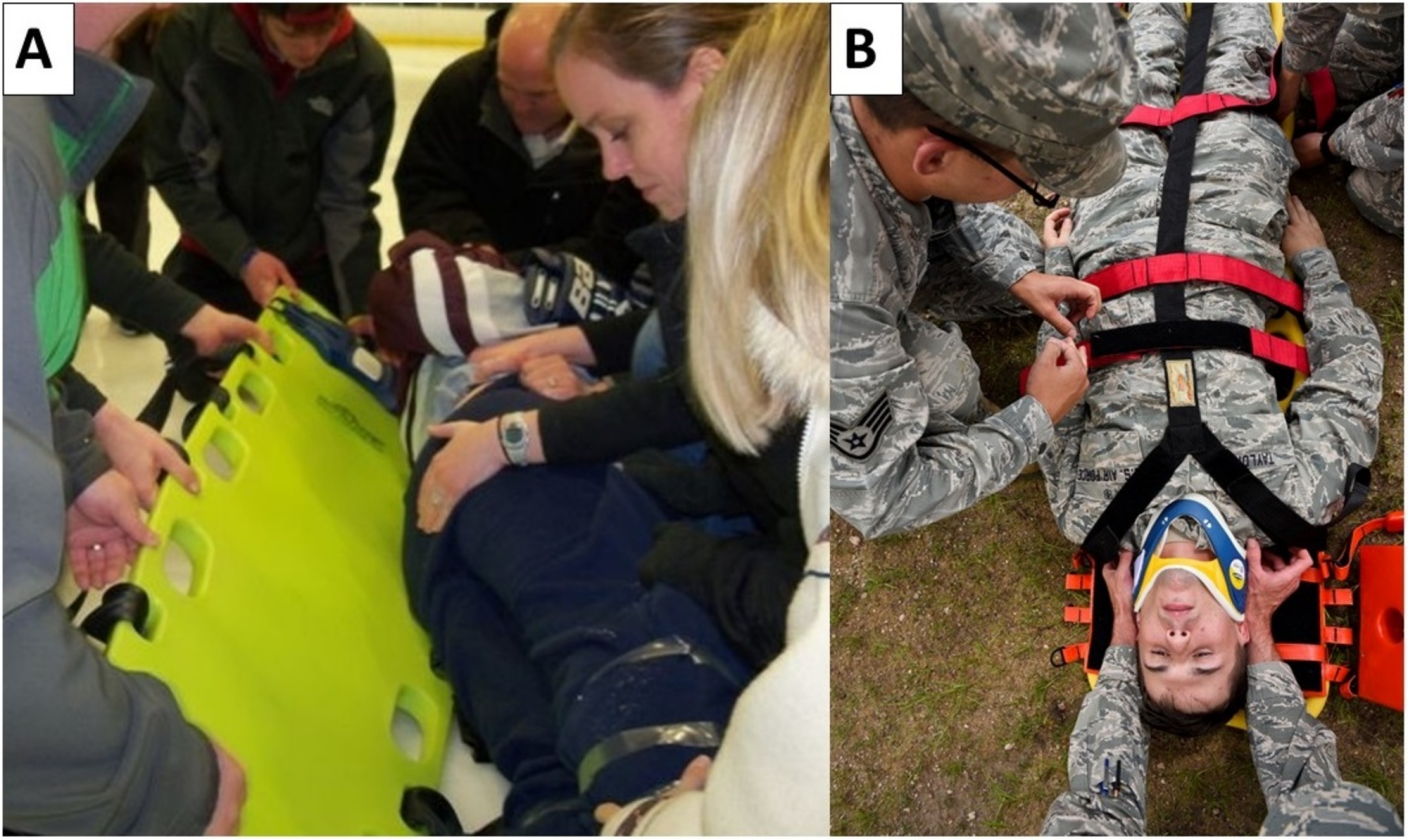
Log-roll techniques Log-roll techniques with in-line neck stabilization (A) and c-collar placement (B).

Once cervical precautions are in place, spinal trauma patients should be placed on backboards until they can be brought to the hospital for a comprehensive cervical spine assessment. Various techniques, such as the log-roll technique for prone patients, have been established to safely place a patient on a backboard (Figure 2) [11-12]. Once the patient is on the backboard, the chest, neck, and helmet must be fully secured to prevent unwanted flexion or extension [8]. The treating providers must then be able to expeditiously transfer the patient to an emergency department for definitive treatment and care.

Due to the uncommon yet potentially fatal scenario of head trauma in hockey games, we aim to create simulation case designed for all emergency medical responders to practice the safe extrication of a collapsed hockey player in a space-restricted environment. Since many commercial sporting events are covered by sports medicine physicians, emergency medicine technicians, and athletic trainers, the authors aim to develop a high-fidelity, sporting event scenario that engages and involves interprofessional audiences simultaneously to support teamwork, problem-solving, and management of available resources. The authors posit that the case has the potential to teach participants to identify, manage, and treat the undifferentiated syncope patient in a sports setting.

## Technical report

Methods

The simulated case was based on a fictional scenario of a hockey player in full gear (including a helmet, shoulder and shin pads, hockey pants, gloves, skates, and hockey jersey) who suffered a face-down collapse within an enclosed bench area shortly after skating off the ice rink without returning to his baseline mental status. The case was jointly written by two simulation-trained emergency medicine physicians, four medical education fellows, and an emergency medicine resident. All facilitators were required to review the literature for sports gear removal in post-head-traumatic events. A simulated patient (i.e., an actor) was integrated into the case to provide for heightened realism. Participants included sports medicine fellows, emergency medicine technicians, and athletic trainers, as these would be the personnel staffing such an event. The simulation took place at a commercial indoor hockey arena.

Equipment

**Figure 3:**
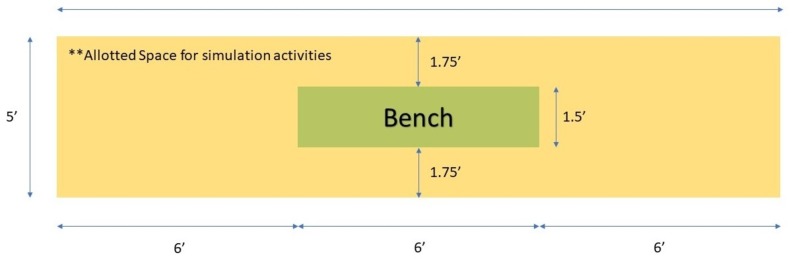
Simulated ice hockey bench blueprints There should be sufficient room for the player to safely collapse in between the wall of the enclosed space and bench. Masking tape or office supplies can be used to mark the designated workspace.

1. Area set up: simulated bench region (Figure 3).

2. Moulage – hockey gear. Required: 1) helmet with faceguard; 2) shoulder pads; 3) hockey pants; 4) sports jersey

3. Emergency medical technician (EMT) equipment: Cervical collar (standard), conventional backboard, portable vital sign monitor, simulated point-of-care testing (i.e., glucose test), and gloves

4. Debriefing space

Personnel/Roles

**Figure 4:**
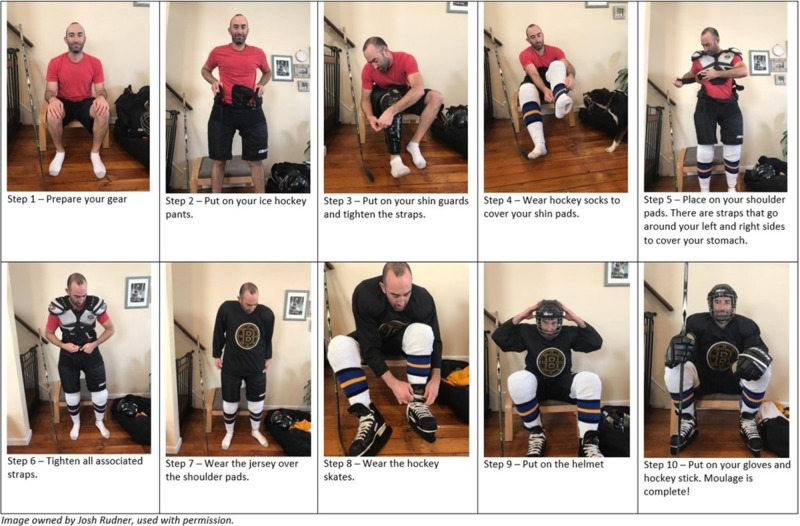
10-step directions to put on an ice-hockey gear

1. Coach – Can be performed by resident/nurse/attending. Outfit: casual, non-player sports attire

2. Patient – Can be performed by resident/nurse/attending. Outfit: hockey gear; we recommend reviewing the guide of donning hockey gear prior to the simulation (Figure 4)

3. Additional hockey players (optional) – Can be performed by resident/nurse/attending. Outfit: sports gear optional, sitting at opposite ends of the bench

Implementation

In order to effectively recreate a confined space to approximate the player’s bench, facilitators can use masking tape to create a simulation zone that is approximately 5’L x 18’W with a removable bench (approximately 1.5’L x 6’W by 2H’) in the center of the area. There should be sufficient room for the player to safely collapse in between the wall of the enclosed space and the bench. Cardboard poster boards can be utilized to create a physical barrier to enhance the experience, although this is not required. Facilitators should be instructed to treat the boundaries as if they are real walls.

**Figure 5:**
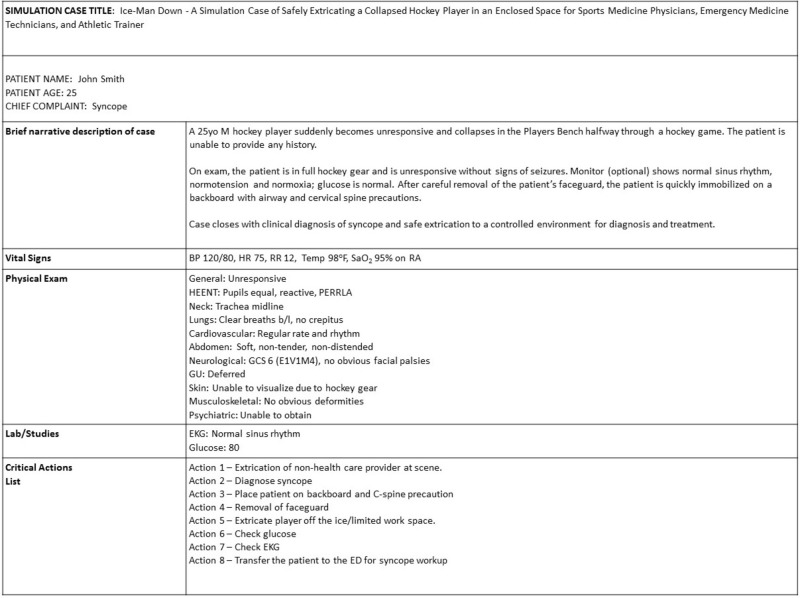
Simulation on ice case flow

Before the case begins, facilitators are advised to first establish the roles of each confederate and provide a preview of the flow of case (Figure 5). The actor playing the injured hockey player should familiarize himself/herself with the appropriate method of donning the hockey gear. All EMT equipment should be stored in an area adjacent to the simulation, which can be easily accessed after a player collapses. Participants should be encouraged to vocalize any interventions, although they should be restricted from performing any invasive procedures to the actor (i.e., intravenous catheter placement or venipuncture). All available laboratory tests should be provided to the learners upon request. The case begins with the player stumbling into the tape-enclosed environment, and safely collapsing onto the ground, immediately adjacent to the player bench. Following the simulation, the participants should complete a bench-side debriefing.

Assessment

Learners will be assessed based on their active participation in both the case scenario and case debriefing. The debriefing will be separated into three components: 1) bench-side teaching and 2) question and answer format.

Bench-side teaching

At the end of the case, the instructor(s)/debriefer(s) should gather information about the delegation of roles and responsibilities while eliciting a case summary from the team leader. The instructor(s) can also request supplemental case information and participant performance, specifically strengths and opportunities for improvement from the team members or observers (if available). The instructor(s) should review the critical action checklist, which includes questions and emphasizes the importance of gathering pertinent patient information and exposing patients for a complete physical examination.

Question and answer session

All learners are encouraged to participate. The debriefing session concludes with each participant sharing one take-home message they drew from the simulation case with the rest of the group.

Results

Participants were able to identify the patient’s decreased level of consciousness and were able to collectively agree on the need to transfer him to a higher level of care. The athletic trainer group reacted quickly, and were the first providers to arrive at the scene; they quickly removed the bench from the enclosed space, performed a rapid primary survey, and directed the removal of the non-health care professionals (i.e., optional players) who were crowding the scene of the accident. The sports medicine physician group elected to focus on the etiologic cause of the patient’s syncope, and interviewed bystanders/witnesses to collect collateral information (i.e., medical, family, and social histories). All three groups recognized the need to maintain airway precaution and cervical spine immobilization in an unresponsive patient who needed to be safely extricated from a public setting; they were unable, however, to reach a consensus on how to rapidly remove the patient’s sports gear, including his helmet, before he was placed on the backboard. The team ultimately elected to keep all of his gear intact, including his helmet and visor and the patient was repositioned with direct in-line stabilization from a prone position to a supine position onto the backboard. The learners discussed the utility of checking a point-of-care glucose level, but ultimately elected to transfer the patient directly to the nearby emergency department after the coach deemed he was stable for transfer.

While all participants in the simulation witnessed the patient stagger and collapse onto the floor, they were unclear of any potential precipitating traumatic events that may have led to his underlying pathology. This required several prompts by the coach confederate to guide their dialogue away from any preexisting trauma. There happened to be a substantial delay, during which participants were not clear about the mechanism of the patient’s fall, and discussed the utility of removing his sports gear and employing cervical spine precautions.

During the debriefing, successive open-ended questions, case summarization, and sharing of take-home-messages demonstrated the participants’ understanding of the clinical and team-based learning objectives.

## Discussion

Our report presents a fictional, yet realistic, case of a hockey player with undifferentiated syncope while participating in a sporting event. The case required learners to work as a team in assessing and managing a hockey player turned patient to ensure the safe stabilization, extrication, and transport of the player to safety in order to be able to further manage him. Syncope, being a rather common complaint encountered by emergency providers and first responders, was thought to be an ideal case to be presented to a multidisciplinary group to attempt to solidify concepts and highlight potential pitfalls in addressing these patients.

The case was performed twice, followed by structured debriefing sessions after each simulation. The authors were able to appreciate noticeable differences between teamwork and time-management skills within the group when the case was repeated. During the second iteration of the case, the team worked more cohesively and effectively. It was also noticed that emergency medicine technicians and athletic training personnel were more likely to take an immediate hands-on approach, while the participating physicians took a more methodical approach to assess possible causes and management strategies.

Overall, the case was well received by all participants, and all participants expressed how the case was pertinent to their daily practice and built upon basic concepts, while introducing several not-so-commonly encountered situations, achieving positive learner reactions (Level 1) and learning (Level 2), per the Kirkpatrick Model. In addition, the debriefing sessions were productive; the authors were able to note an improvement in teamwork and communications skills within the group when the case was repeated.

Limitations

During this simulation exercise the group of participants was too large. This led to a small number of participants taking a leadership role, while others were more passive during the case and the debriefing. In such a large setting (i.e., 10-20 participants), it may be challenging to have delineated roles for all participants. The simulation was also performed in a hockey arena, which may not be generalizeable to other institutions and, therefore, limits fidelity. This can easily be addressed, however, with appropriate patient/player moulaging, border regulations, and confederate preparation (as described in the implementation section).

## Conclusions

As hockey continues to gain popularity, this simulation case offers athletic trainers, sports medicine physicians, and first responders the unique opportunity to deliberately practice the skills required to manage syncope in hockey players, as well as safely and effectively extricate injured players from unpredictable, space-limiting sporting event settings.
